# Determinants of undergraduate medical students’ satisfaction with clinical supervision: A cohort study in a longitudinally structured sixth year clinical placement

**DOI:** 10.1007/s00508-024-02477-4

**Published:** 2024-12-06

**Authors:** Michaela Wagner-Menghin, Angelika Hofhansl, Lena Bach, Anna-Maria Mayer, Anita Rieder, Gerhard Zlabinger

**Affiliations:** 1 Division of Social Psychiatry, Department of Psychiatry and Psychotherapy, Medical University of Vienna, 1090 Vienna, Austria; 2 Comprehensive Center for Clinical Neurosciences and Mental Health, Medical University of Vienna, 1090 Vienna, Austria; 3 Teaching Center, Medical University of Vienna, Vienna, Austria; 4 Institute of Empirical Educational Sciences, Catholic University of Eichstätt-Ingolstadt, Eichstätt, Germany; 5 Vice-rectorate for Education, Medical University of Vienna, Vienna, Austria; 6 Center for Public Health, Medical University of Vienna, Vienna, Austria

**Keywords:** Clinical supervision, Medical education, Undergraduate students, Students’ perspective, Workplace-based learning

## Abstract

**Background:**

Work satisfaction is associated with fewer employee turnover intentions, increased job engagement and interest, and has a greater impact on employee well-being than environmental factors, such as workload. In workplace learning, clinical supervisors promote student satisfaction by meeting students’ supervision needs in providing safe practice opportunities, training, and guidance in the social field. To quantitatively investigate this relationship, we proposed a supervision deficit index as a measure of learner-centered supervision received and explored its correlation with satisfaction in workplace learning.

**Method:**

In total, 1017 Austrian medical students (2015–2017) in year 6 selected the 5 most helpful supervisory activities (from 26 options) and rated their experience levels of these activities during surgery and internal medicine placement. A supervision deficit index was then created (range 0–3; 0 = no deficit).

**Results:**

Students with no, minor or moderate supervision deficits reported higher overall satisfaction with their placements than those experiencing considerable deficits. Students’ gender, clinical experience, hospital size, placement year, and clinical field did not influence the relationship. The deficit index’s psychometric qualities were good. Training activities supporting competence, such as discussing patients, planning disease management, and practicing skills, were selected more often than activities supporting autonomy, such as an appropriate level of clinical duties, and social relatedness.

**Discussion:**

Students favored competence support. Highlighting the importance of autonomy support to students and encouraging supervisors to engage in learner-centered supervision may improve the supervision experience and work satisfaction for both. The deficit index can be used to evaluate the effects of such interventions.

## Introduction

A clinical supervisor acts as a gatekeeper by carefully steering students’ access to tasks to ensure the quality of professional services provided by trainees to patients, as a trainer to develop their competence, and as a mentor to support learning in the workplace [1]. Although the workplace is theoretically an authentic learning environment for complex professional skills [2], it does not automatically provide an ideal learning setting for medical students, and learning experiences vary markedly between students and clinics [3, 4]. A curricular structure with defined learning objectives for participation in the clinical team and patient care under close supervision can promote learning in the clinical workplace [1]. Nevertheless, even in best-practice workplace learning programs students express concerns about making mistakes, staff neglect, and poor adjustment to the clinical setting [5]. Only about one third of undergraduate medical students (UGMS) report being very satisfied with their clinical supervision, around 40% report achieving their desired progress during the placement, and only about 50% would recommend their placement department to a friend [6]. From the program providers’ perspective, this lack of learners’ positive subjective reactions should be a cause for concern. An association between positive subjective reactions to working or learning and favorable employee outcomes [7–9] has been established. These outcomes include less exhaustion, fewer turnover intentions [7, 9], increased job engagement [7], or interest in a clinical field [10]. Even the absolute clinical workload remains unidentified as “the core issue” in the lack of well-being among obstetrics and gynecology trainees and faculty physicians. Instead, “work satisfaction influences well-being more than workplace environment” [8, p. 4]. As such, evaluating subjective reactions beyond objective learning outputs, behavior changes, and long-term outcomes has been an important pillar of training program evaluation since Kirkpatrick suggested this fourfold approach for program evaluation in 1959 [11].

To improve satisfaction with supervision, placement duration [12], schedule feasibility, learning material quality, examination fairness [13], and opportunities for participation in clinical activities [14] have been investigated as determinants of satisfaction. Still, supervisor engagement contributes the most to student satisfaction with placement [15]. Good supervisors can alleviate students’ concerns and foster their learning and sense of belonging [16, 17]. Consequently, parallels between patient-centered care and learner-centered supervision have been drawn [13, 18]. Thus, we propose developing a quantitative measure expressing students’ experiences of how their supervision needs were met based on an empirically tested theory from motivation psychology.

According to self-determination theory (SDT; [19]), a person needs to experience competence, autonomy, and social relatedness in their social environment to be motivated, satisfied, and to perform well. A central assertion of SDT, as applied to clinical supervision, is that supporting students’ basic psychological needs, here coined learner-centered supervision, promotes motivation, learning, and satisfaction. To expand our understanding of the mechanisms of learner-centered supervision and satisfaction in the workplace, we will use the lens of SDT to introduce a measure to quantify students’ learning experience, the supervision deficit index (SDI). The index is based on supervisory activities that students indicate as being most helpful for their learning. The discrepancy between desired and received activities reflects the individually experienced supervision deficit.

Consequently, we will address the following question: How do students’ perceptions of a deficit in learner-centered supervision contribute to explaining general satisfaction with supervision? Based on SDT, it is hypothesized that experiencing a deficit negatively impacts general satisfaction.

## Methods

### Participants and procedure

The study participants were UGMS from the Medical University of Vienna/Austria completing year 6 in 2015, 2016, and 2017 (*n* = 1712), who completed both compulsory placements (internal medicine and surgery) in hospitals in Austria (*n* *=* 1017) and provided data for at least one of them (*n* = 529) during the year 6 evaluation week. Returning the anonymously completed paper-based material signified consent to participate.

### Setting

The integrated organ-based undergraduate medical curriculum includes placements in years 3 and 4 (12 weeks clinical traineeship, *Famulatur*), five 6‑week and two 3‑week structured placements in year 5 (clinical classes, *Klinischer Unterricht*). For the longitudinally structured clinical placement in year 6, (clinical practical year, *Klinisch-Praktisches Jahr*), students apply for positions offered by more than 100 contracted teaching hospitals in Austria and other teaching hospitals abroad. The three periods (surgery, internal medicine and elective topic, 16 weeks each) are organized following the basic principles of an effective supervision framework [1] and are supported by a logbook [20]. The completion sequence for the three periods is based on students’ preferences and position availability. The hospital’s teaching coordinator pairs each accepted student with a clinical supervisor (resident or senior doctor) for a 1:1 relationship during the given period. Supervisors participate in training before (or on) being paired, where they spend 4h learning about legal, organizational, and educational duties, such as gatekeeping, training, and mentoring, scheduled feedback meetings, structured assessments and documentation in the logbook.

### Measures

#### Independent variable

##### Deficit in learner-centered supervision.

Supervision deficit was assessed based on a 26-item list of supervisory activities, including gatekeeping, training, and mentoring activities. Responsible faculty members (anonymized) translated items out of a published supervisory activity list (Grant et al. 2003 [21]) into German, categorized them into the three supervisory roles and added six items to extensively cover the mentoring role (Table 1). Students were instructed to select five activities they favored as particularly helpful for their learning and indicated supervisors’ engagement in each of the activities using a 4-point rating scale (To what extent has your clinical supervisor engaged in the following activities? (1) “not at all,” (2) “to a small extent,” (3) “to a relevant extent,” and (4) “to a full extent”). The supervision deficit (SD) for each favored activity was calculated as follows: SD_(activity)_ = 4 − rating_(supervisor engagement in this activity)_. Not all students selected five activities as instructed, so data were included as valid for counts between 1 and 14 activities, and the person-wise mean was calculated as the SDI. A value of 0 indicates that all favored supervisory activities have been fully received. The value 1 [> 0–1] indicates a minor deficit, and 2 [> 1–2] indicates a moderate deficit. The value 3 [> 2–3] constitutes a considerable deficit, indicating that the majority or all the favored supervisory activities were not received at all.

**Table 1:** Students’ perceived helpfulness of supervisory activities and rating scale model item statistics for the supervision deficit index

Supervisory activities grouped by supervisory role	Perceived helpfulness of supervisory activities Relative frequencies^1^		Quality of items Rating scale model item statistics		
	Internal medicine	Surgery	Item location^2^	MSQ Infit^3^	MSQ Outfit^3^
*Gatekeeping*					
Ensure patient safety	0.09	0.09	1.512	0.856	1.242
Ensure the safety of the trainee	0.09	0.08	1.472	1.342	1.045
Ensure appropriate level …	0.15	0.18	0.204	0.955	0.915
Provide feedback through appraisal	0.06	0.05	−0.647	1.210	1.242
Monitor the trainee’s performance	0.02	0.04	0.906	***0.541***	0.668
Discuss/review the … supervision	0.10	0.09	−0.290	1.018	1.117
Address successes/problems …	**0.25**	**0.22**	−0.290	0.748	0.720
*Training*					
Discussing individual patients	**0.58**	**0.52**	0.000	0.856	0.755
Discuss (away …) management …	**0.51**	**0.40**	−0.001	0.764	0.699
Teach … techniques, procedures	**0.47**	**0.53**	0.164	0.861	0.805
Bedside teaching	**0.42**	**0.34**	0.082	0.935	0.983
Provide informal feedback	**0.28**	**0.25**	−0.391	0.818	0.844
Plan learning	0.10	0.09	−1.458	0.814	0.918
Develop presentation skills	0.07	0.05	−0.674	***1.996***	***2.002***
Encourages critical thinking	**0.23**	0.16	0.205	0.692	0.603
*Mentoring*					
Share professional experience*	**0.20**	**0.21**	1.077	***0.425***	***0.483***
Show alternatives/solutions …*	0.08	0.08	−0.654	0.763	0.761
Gives orientation … context*	0.07	0.10	0.128	0.910	0.994
Gives emotional support*	0.05	0.05	−0.286	1.017	1.187
Develop interpersonal skills	0.07	0.05	0.014	0.707	***0.592***
Develop teamwork skills	0.12	0.13	−0.308	1.018	1.024
Develop communication skills	0.03	0.03	0.036	0.679	0.757
Guide personal/professional dev	0.11	0.19	−0.157	***0.518***	***0.558***
Support personal/professional dev	0.15	0.10	−0.289	0.663	***0.577***
Counsel career development*	0.09	0.15	−0.659	1.204	1.297
Positive role model*	0.13	0.15	0.440	0.863	0.647

*MSQ* Mean Square

*Supervisory activities not included in Grant et al. 2003 [21]

^1^Students selected 3–7 activities they perceived as particularly helpful for their learning. Relative frequencies ≥ 0.20 are in bold (arbitrary set cut-off value), *n *(internal medicine) = 502, *n *(surgery) = 421

^2^Item location on the latent supervision deficit dimension, higher values indicate that persons with a higher location value have a higher likelihood of experiencing a deficit for the activity. Estimation included 873 ratings with more than 2 selected activities, 733 missingness patterns were observed. Person location parameters and standard errors for 604 ratings with sufficient variance in their response vector were estimated (*M* = 0.55, *SD* = 4.797, min. = −2.12, max. = 4.80), the high number of missingness patterns did not allow extrapolation of person parameters for the remaining ratings

^3^Values > 1.4 (less predictability, underfit) and values < 0.6 (more predictability, overfit) in the data as compared to the model [22, p. 179] are in bold italic

#### Control variables

##### General clinical experience.

Students entering internal medicine [23] or surgery placements [24] have been found to benefit in their learning from previous workplace learning experience, which prompted us to include this control. Students indicated the period in their trajectory (first, second, and third) when they completed the respective placement.

##### Hospital size.

Previous empirical studies mention a more inviting educational atmosphere in smaller community hospitals [25] or smaller teaching hospitals [26]. Higher patient satisfaction is linked to smaller hospitals due to higher person-centeredness [27]. Hospital size was coded using the official information about the number of beds and categorized as follows to build groups of approximately equal size: 1 (up to 350 beds), 2 (351–650 beds), 3 (651–1000 beds), 4 (more than 1000 beds). The majority but not all the smaller hospitals are contracted teaching hospitals; the majority but not all of the larger hospitals are university hospitals.

##### Other control variables.

Gender, year of graduation, and clinical field of placement were also used as controls.

#### Dependent variable

##### Satisfaction with supervision during placement.

A single item measure was used to rate general satisfaction (7-point rating scale; −3 to 3) with clinical supervision in each placement. Values were transformed to 0–6 for analysis.

### Statistical analysis

#### Reliability and item quality

The reliability of the SDI was determined using a rating scale model (RSM). The model provides estimates of person and item location, with rating category thresholds fixed across all items. Estimation can be performed using a data matrix with multiple missingness patterns caused by the procedure. For further item development, infit and outfit mean squares (I/O MSQ) are interpreted using the rule of thumb for rating-scale data: MSQ values > 1.4 indicate less predictability (underfit) and values < 0.6 more predictability in the data as compared to what is predicted by the model (overfit). To identify which activities the students desired the most within each placement, relative selection frequencies were calculated for each activity.

#### Contribution of deficit in learner-centered supervision in explaining general satisfaction with supervision

To evaluate the supervision deficit influence on general satisfaction while controlling for the influence of general clinical experience, clinical field of the placement, gender, and hospital size, generalized estimating equations were used. This multilevel regression-based analysis approach accounts for clustered data, such as family data, or data from repeated measurement. Initial regression parameters were estimated using a generalized linear model that ignored clustering. In the second step, the standard error estimates were adjusted using an initially defined correlation structure. Wald‑χ^2^ tests were reported to indicate predictors with a significant influence on the outcome variable. To compare the fit of different models, we used the quasi-likelihood information criterion (QIC) and its corrected value (QICC), with smaller values indicating better model fit. Descriptive statistics for the SDI, control and outcome variables are given in the Appendix.

IBM SPSS Statistics for Windows, Version 28.0, (IBM Corp., Armonk, NY, USA), with the GENLIN procedure, and eRm [28] were used. The Medical University of Vienna/Austria Board for Privacy Protection reviewed the study protocol and granted permission to process the data.

## Results

### Reliability and item quality

The extent of students’ preference for each supervision activity, as expressed by the relative frequencies of selecting an activity as helpful, ranged from 0.02 to 0.58 for the internal medicine placement (*n* = 502) and from 0.03 to 0.53 for the surgery placement (*n* = 424). A clear preference emerged for teaching activities over gatekeeping or mentoring activities (Table 1). The RSM item parameters covered 3 units of the latent supervision deficit dimension (−1.46 to 1.51). This indicates that reliable measurement for subjects experiencing high, medium, and low levels of supervision deficit is possible with the available items. Fit indices indicated that all but five items contributed to determining the subjects’ location on the supervision-deficit dimension, as required by the RSM (Table 1). Within the subsample with an estimable person parameter, an empirical reliability of 0.735 emerged, which follows the theoretically expected reliability for rating scales with four categories used with 3–7 perfectly functioning items [29].

### Contribution of deficit in learner-centered supervision in explaining general satisfaction with supervision

In the study three models (model 1, 2, 3) were used to address how students’ perception of a deficit in learner-centered supervision contributes to explaining general satisfaction with supervision and two competing models (model C, Ca) were used to explore the predictive value of the newly introduced SDI. The significant Wald test of model 1 (SDI as predictor) indicates that differences in experiencing supervision deficit contribute to differences in general satisfaction (Table 2). The estimated means for the no, minor, or moderate deficit categories (*M* = 5.78, 5.27, 4.11) indicate that students experiencing the three categories show higher general satisfaction with their clinical placement than those experiencing a considerable supervision deficit (*M* = 2.18).

**Table 2:** GEE models 1, 2 and 3, competing models C and Ca, effect and goodness of fit statistics

		Effect			Goodness of fit	
		Wald‑χ^2^	Df	Sig. ^1^	QIC	QICC
*Model 1*	(Constant)	4979.072	1	**<** **0.001**	111.937	118.957
	Supervision deficit index	364.214	3	**<** **0.001**		
*Model 2*	(Constant)	4694.536	1	**<** **0.001**	112.475	128.783
	Supervision deficit index	346.784	3	**<** **0.001**		
	General clinical experience	1.584	2	0.453		
	Hospital Size	1.036	3	0.793		
*Model 3*	(Constant)	4358.476	1	**<** **0.001**	112.765	138.486
	Supervision deficit index	335.530	3	**<** **0.001**		
	General clinical experience	1.607	2	0.448		
	Hospital Size	1.309	3	0.727		
	Gender	3.087	2	0.214		
	Year	0.700	2	0.705		
	Clinical field	1.821	1	0.177		
*Model C*	(Constant)	19,070.653	1	**<** **0.001**	164.462	208.620
	General clinical experience * Hospital size * clinical field	62.955	23	**<** **0.001**		
*Model Ca*	(Constant)	4756.706	1	**<** **0.001**	113.344	163.294
	General clinical experience * Hospital size * clinical field	28.488	23	0.198		
	Supervision deficit index	335.848	3	**<** **0.001**		

*GEE* generalized estimating equations, *Constant* y-intercept in the equation = expected value of the dependent variable when all independent variables are equal to zero.

^1^ values ≤ 0.05 are in bold, *n* = 923, 528 subjects, *QIC/QICC* quasi-likelihood under the independence model criterion/corrected

Model 2 explored how including the predictors general clinical experience (three categories) and hospital size (four categories) changed the supervision deficit impact on general satisfaction. Adding these control variables did not generally improve the model, as indicated by the similar values for QIC and QICC. In addition, neither of the two predictors changed the dominant influence of the SDI in explaining general satisfaction, as indicated by the significant Wald test for the SDI and the insignificant ones for clinical experience and hospital size. Model 3 incorporated gender (undefined, male and female), graduation year (2015, 2016, and 2017), and clinical field (internal medicine, and surgery) as predictors. Adding these control variables did not improve the model compared to models 1 and 2, as indicated by similar values for QIC and QICC for both models; in addition, the Wald tests for those new predictors did not reach significance (Table 2).

Given the lack of significant main influences of the control variables above the SDI, we systematically explored whether interactions between hospital size, general clinical experience, and clinical field, explained differences in satisfaction. The interaction of hospital size, clinical experience, and clinical field emerged to explain satisfaction (model C); however, the QIC and QICC values of model C markedly exceeded those of model 1. When the SDI was added to the model to build model Ca, the interaction term became insignificant, and the QIC and QICC values dropped, indicating that model Ca had a better fit than model C (Table 2).

## Discussion

Young and inexperienced UGMS are in need of high-quality supervision [16] during their clinical placement, as their study behavior is strongly influenced by supervision quality. To help define high-quality supervision, we draw on the SDT basic psychological needs concept. Meeting students’ supervision needs is an important aspect of supervision quality, as seeing one’s needs met strongly impacts one’s satisfaction. The extent and quality of supervisor behavior require consideration when evaluating students’ subjective supervision experiences [30]. Expanding this idea, we present an SDI to represent the extent to which students’ supervision needs were met in a single measure. With the currently implemented choice procedure, a reliability of 0.735 was observed, with satisfactory psychometric quality for most of the 26 items.

Following the SDT’s basic psychological needs concept, experiencing learner-centered supervision was confirmed as the main predictor of satisfaction with clinical placement. General clinical experience, clinical field, hospital size, and gender did not contribute to satisfaction above the SDI. Our results empirically reinforce previous opinions [18]. Structuring a teaching encounter with a student as one would structure a patient encounter, with a strong focus on personalizing the encounter, inviting a shared presence, engaging students, checking their understanding, and building trust is the key to the “quality of interactions between residents and medical students*” *[31, p. 348]. Consistent with our findings are results on favorable resident teacher attributes [32]: Besides “having a strong knowledge base,” “tailoring teaching to learner’s level”, and being “approachable” are the top favored teacher attributes indicated by over 70% of students out of five Canadian medical schools. Our findings corroborate those reporting that fulfilment of medical students’ basic psychological needs reduces their stress [33].

On average, UGMS favored the same seven supervisory activities in both clinical fields, where five are training activities, one is a gatekeeping and one a mentoring activity. This result seems to corroborate the reporting that satisfaction of competence, but not autonomy or relatedness, predicted increased students’ resilience [34]. About half of the UGMS favored the two training activities, “discuss individual patients” and “discuss away from the bedside”, which postgraduate medical students and their supervisors also indicated as the most helpful [21]. The other five activities were regarded as most helpful by an even smaller share of UGMS in our study and also regarded as less helpful during postgraduate training.

As such, the following practical implications for clinical supervisors and program providers can be derived: (a) students’ supervision needs are generally similar between internal medicine and surgery placements, despite subtle differences, but show high variability between students. Although a one size fits all approach to supervision is nonexistent, identifying opportunities for participation in the daily clinical routines and allowing attending UGMS to experience the three most popular training activities regularly during their stay is a good strategy to prepare oneself as a clinical supervisor. (b) Student factors such as their prior general clinical experience and gender, or external factors, such as hospital size and clinical field, do not explain satisfaction above the SDI in this study. Still, according to a previous study students’ supervision expectations seem to be shaped by prior experiences [35]. A successful supervision approach requires both parties to understand each other’s expectations, needs and resources and to respect boundaries. Supervisors engaging in good student-onboarding practice explore students’ wishes and expectations. They also should openly emphasize their responsibility of gatekeeping to provide training opportunities for students and their struggle to balance patients’ and students’ needs [1]. It may be encouraging for supervisors to know that approximately two third of UGMS report their supervisors to engage fully in gatekeeping activities such as ensuring student and patient safety [35]. In addition, outlining how students can and should actively contribute to the supervisory process, given the boundaries of the respective setting, contributes to understanding each other. (c) Program providers seeking to evaluate UGMS satisfaction with their placement might use a group level SDI to monitor students’ perceptions of learner-centered supervision while further gaining insights into students’ supervision preferences and chances thereof. This approach would be beneficial for evaluating the effect of faculty development on clinical supervisors, such as sharing and refining best-practice examples.

## Limitations

Privacy protection precluded data collection at the department level in the hospitals. Data could also only be collected after all three parts of the clinical placement had been completed. Despite tailoring the paper-based survey procedure to the time available, many students did not follow the instructions to provide five favorites. Thus, more unique missingness patterns with 0 or perfect scores emerged, for which person location parameters could neither be estimated nor extrapolated. Optimizing and shortening the set of activities will facilitate the completion of the choice procedure as instructed. Future studies might consider using our item quality results to shorten the list of activities and modify or abandon the choice procedure to reduce the number of observed missingness patterns. Studies should either control for sequence effects when collecting data after completing all parts of the clinical placement or strive to collect data on supervisory experience shortly after each part of the training. Providing the survey online may contribute to all suggestions for improvement.

## Conclusion

Providing learner-centered supervision promotes students’ motivation and thus their learning [8]. With the SDI, we presented in principle how to reliably and validly quantify the lack of learner-centered supervision, which might be a risk factor for falling short in learning. Using the index, we showed that addressing and fulfilling students’ supervision needs is more important for student satisfaction with their clinical placement than context variables such as hospital size or their previous general clinical experience. We replicated the results on UGMS, who, like postgraduate trainees, preferred competence support above autonomy and relatedness support in workplace-based learning. With the SDI, the quantitative evaluation of interventions targeting improving students’ and supervisors’ satisfaction with supervision is possible. Clinical supervisors and program providers might be interested in considering how their students and potentially future colleagues experience supervision above evaluating the students’ learning output [11], as those experiences influence their career choice [10, 36].

## Data Availability

The data that support the findings of this study are available from the corresponding author upon reasonable request.

## Appendix

**Table 3:** Descriptive statistics—Model parameters

	Internal medicine *n* = 502		Surgery *n* = 421		Total *N* = 923	
	Count	%	Count	%	Count	%
*Supervision deficit index*						
0 no deficit	143	29	137	33	280	30
1 minor deficit	203	41	163	39	366	40
2 moderate deficit	100	20	74	18	174	19
3 considerable deficit	55	11	47	11	102	11
*Clinical experience*						
Level 1	182	36	134	32	316	34
Level 2	195	29	162	39	357	39
Level 3	125	25	125	30	250	27
*Gender*						
1	260	52	203	48	463	50
2	223	45	201	48	424	46
Undisclosed	18	4	17	4	35	4
*Year*						
2015	174	35	151	36	325	35
2016	151	30	124	30	275	30
2017	176	35	146	35	322	35
*Hospital size*						
Beds ≤ 350	84	17	73	17	157	17
Beds 351–650	118	24	120	29	238	26
Beds 651–1000	169	34	112	27	281	31
Beds > 1000	130	26	116	28	246	27
	***M***	***SD***	***M***	***SD***	***M***	***SD***
*Satisfaction (0/6)*	4.90	1.488	4.82	1.564	4.86	1.523

*Level 1* Year 3, 4 and 5 placements. *Level 2* (Level 1) + 16 weeks from the first period of year 6 placement. *Level 3* (Level 1) + (Level 2) + 16 weeks from second period of year 6 placement
